# Breast cancer risk markedly lower with serum 25-hydroxyvitamin D concentrations ≥60 vs <20 ng/ml (150 vs 50 nmol/L): Pooled analysis of two randomized trials and a prospective cohort

**DOI:** 10.1371/journal.pone.0199265

**Published:** 2018-06-15

**Authors:** Sharon L. McDonnell, Carole A. Baggerly, Christine B. French, Leo L. Baggerly, Cedric F. Garland, Edward D. Gorham, Bruce W. Hollis, Donald L. Trump, Joan M. Lappe

**Affiliations:** 1 GrassrootsHealth, Encinitas, California, United States of America; 2 Department of Family Medicine and Public Health, University of California San Diego, La Jolla, California, United States of America; 3 Medical University of South Carolina, Charleston, South Carolina, United States of America; 4 Inova Schar Cancer Institute, Falls Church, Virginia, United States of America; 5 Department of Medicine, Creighton University, Omaha, Nebraska, United States of America; University of Tennessee Health Science Center, UNITED STATES

## Abstract

**Background:**

While numerous epidemiologic studies have found an association between higher serum 25-hydroxyvitamin D [25(OH)D] concentrations and lower breast cancer risk, few have assessed this association for concentrations >40 ng/ml.

**Objective:**

To investigate the relationship between 25(OH)D concentration and breast cancer risk across a broad range of 25(OH)D concentrations among women aged 55 years and older.

**Methods:**

Analyses used pooled data from two randomized clinical trials (N = 1129, N = 2196) and a prospective cohort (N = 1713) to examine a broad range of 25(OH)D concentrations. The outcome was diagnosis of breast cancer during the observation periods (median: 4.0 years). Three analyses were conducted: 1) Incidence rates were compared according to 25(OH)D concentration from <20 to ≥60 ng/ml (<50 to ≥150 nmol/L), 2) Kaplan-Meier plots were developed and 3) multivariate Cox regression was used to examine the association between 25(OH)D and breast cancer risk using multiple 25(OH)D measurements.

**Results:**

Within the pooled cohort (N = 5038), 77 women were diagnosed with breast cancer (age-adjusted incidence: 512 cases per 100,000 person-years). Results were similar for the three analyses. First, comparing incidence rates, there was an 82% lower incidence rate of breast cancer for women with 25(OH)D concentrations ≥60 vs <20 ng/ml (Rate Ratio = 0.18, *P* = 0.006). Second, Kaplan-Meier curves for concentrations of <20, 20–39, 40–59 and ≥60 ng/ml were significantly different (*P* = 0.02), with the highest proportion breast cancer-free in the ≥60 ng/ml group (99.3%) and the lowest proportion breast cancer-free in the <20 ng/ml group (96.8%). The proportion with breast cancer was 78% lower for ≥60 vs <20 ng/ml (*P* = 0.02). Third, multivariate Cox regression revealed that women with 25(OH)D concentrations ≥60 ng/ml had an 80% lower risk of breast cancer than women with concentrations <20 ng/ml (HR = 0.20, *P* = 0.03), adjusting for age, BMI, smoking status, calcium supplement intake, and study of origin.

**Conclusions:**

Higher 25(OH)D concentrations were associated with a dose-response decrease in breast cancer risk with concentrations ≥60 ng/ml being most protective.

## Introduction

Breast cancer is the most common non-skin cancer in women [1]. More than 252,000 new cases of female breast cancer and 40,600 deaths were projected to occur in 2017 in the United States [1]. While more early detection and improvements in treatment have reduced the mortality rate, there has been no reduction in the incidence of breast cancer in the past 20 years [2]. Identifying and implementing effective primary prevention strategies could reduce breast cancer incidence rates.

Epidemiologic studies by Gorham et al. [3,4] and Garland et al. [5] were the first to propose that vitamin D prevents breast cancer. Since then, the mechanisms by which vitamin D might prevent the development and growth of breast cancer have been well documented [6] and numerous epidemiologic studies have found an association between higher serum 25-hydroxyvitamin D [25(OH)D] concentrations, the physiological measure of vitamin D status, and a lower risk of breast cancer [7–18]. However, few studies have assessed this association in concentrations >40 ng/ml [7,8].

The objective of this analysis was to investigate the relationship between 25(OH)D concentration and breast cancer risk across a broad range of 25(OH)D concentrations among women aged 55 years and older. Data from two randomized clinical trials (RCT) and a prospective cohort study were pooled: the 2007 Lappe et al. cohort (RCT, median 25(OH)D = 31 ng/ml, N = 1129) [19,20], the 2017 Lappe et al. cohort (RCT, median 25(OH)D = 36 ng/ml, N = 2196) [21], and the GrassrootsHealth cohort (prospective cohort study, median 25(OH)D = 49 ng/ml, N = 1713) [20]. This pooled cohort provided a larger sample size for improved statistical power and allowed for analysis across a broad range of 25(OH)D concentrations that would otherwise not have been possible due to the lack of a sufficient number of participants with 25(OH)D concentrations higher than 40 ng/ml.

## Materials and methods

Women in the 2007 Lappe et al. cohort (hereafter termed 2007 Lappe cohort) participated in a four year, population-based, double-blind, placebo-controlled trial of vitamin D and calcium supplementation in a 9-county area in Eastern Nebraska. Participants were randomly assigned to: 1) calcium plus vitamin D3 (1400–1500 mg/day of calcium plus 1100 IU/day of vitamin D3), 2) calcium (calcium as mentioned previously plus vitamin D placebo), or 3) control (calcium and vitamin D placebos). This trial was registered at clinicaltrials.gov as NCT00352170.

In another study, women in the 2017 Lappe et al. cohort (hereafter termed 2017 Lappe cohort) participated in a four year, population-based, double-blind, placebo-controlled trial of vitamin D and calcium supplementation in a 31-county area in Eastern Nebraska. Participants were randomly assigned to: 1) intervention (1500 mg/day of calcium and 2000 IU/day of vitamin D3) or 2) control (calcium and vitamin D placebos). This trial was registered at clinicaltrials.gov as NCT01052051.

For both Lappe cohorts, inclusion criteria included women aged 55 years or older who were free of known cancer at enrollment and within the prior 10 years. As described previously [19–22], supplement intake by bottle weight and health status were assessed at 6-month intervals. Medical records were examined to confirm reports of cancer diagnosis and ascertain diagnosis date. Participants who did not complete at least two health assessments were excluded from this study because of lack of prospective data. Serum 25(OH)D concentrations were measured at enrollment and annually thereafter using radioimmunoassay (IDS Radioimmunoassay (RIA) kit, Fountain Hills, AZ for the 2007 cohort and Liaison^®^ Analyzer, Diasorin, Stillwater, MN for the 2017 cohort) at the Creighton University Osteoporosis Research Center Laboratory (Omaha, NE). The intra-assay coefficient of variation was 5% for IDS RIA and 5% for Liaison^®^. Additionally, the Creighton Laboratory participates in the Vitamin D External Quality Assessment Scheme (DEQAS) with findings on test samples regularly close to the international mean. Detailed descriptions of the Lappe trials and results of other outcomes can be found elsewhere [19–22]. All participants provided written informed consent and the studies were approved by the Creighton University Institutional Review Board (Omaha, NE).

Women in the GrassrootsHealth cohort participated in a prospective population-based cohort study run by a non-profit public health research organization. Voluntary participants, who reside in 57 countries worldwide (91% in the United States or Canada) submitted home blood spot 25(OH)D test kits and completed online health questionnaires. There were no exclusion criteria nor any requirements related to 25(OH)D concentration or supplement intake dose. Participants included both genders and a wide range of ages; however, only female participants aged 55 years or older who were free of known cancer at enrollment and within the prior 10 years who completed at least two health assessments were included in this pooled analysis to match the inclusion criteria of the Lappe cohorts. As described previously [20], cancer diagnosis dates and cancer type were reported as were average daily calcium supplement intake, age, smoking status, height, and weight. Serum 25(OH)D concentrations were determined by analysis of dried blood spot test kits using liquid chromatography-mass spectroscopy (LC-MS/MS) by ZRT Laboratory (Beaverton, OR) or Purity Laboratory (Lake Oswego, OR). The intra-assay coefficient of variation was 9% for ZRT and 5% for Purity. Additionally, the ZRT and Purity assays have been validated against the DEQAS LC-MS/MS consensus group (*R*^2^ values of 0.998 and 0.994 respectively). LC-MS/MS with dried blood spot cards has been validated against the radioimmunoassay method (*R*^2^ value of 0.91 and a slope not different from 1.0) [23]. All participants provided informed consent and the study was approved by the Western Institutional Review Board (Olympia, WA).

Overall, this analysis included 1129 women from the 2007 Lappe cohort (median follow-up time, 4.0 years), 2196 women from the 2017 Lappe cohort (median follow-up time, 4.0 years), and 1713 women from the GrassrootsHealth cohort (median follow-up time, 1.9 years) (pooled cohort N = 5038; median follow-up time, 4.0 years).

### Statistical methods

Demographic characteristics were summarized and comparisons between cohorts were performed using Kruskal-Wallis tests for age, BMI, calcium supplement intake (study and non-study combined for Lappe cohorts), and serum 25(OH)D. The chi-square test was used for smoking status. While data was collected for all types of cancer diagnoses, the outcome of interest for this current study was the diagnosis of breast cancer (invasive or in situ) during the observation periods. Age-adjusted breast cancer incidence rates were calculated using direct standardization to the 2010 US population [24].

Three analyses were conducted to investigate the relationship between 25(OH)D concentration and breast cancer. First, breast cancer incidence rates and their 95% confidence intervals (95% CI) were calculated for successive 20 ng/ml strata of serum 25(OH)D concentration from <20 to ≥60 ng/ml using a moving average method [25–27] to assess incidence trends across the range of 25(OH)D. A rate ratio (incidence density ratio) for <20 vs ≥60 was calculated to compare incidence rates.

Second, Kaplan-Meier curves comparing the proportion of breast cancer-free participants by 25(OH)D group were developed to estimate breast cancer-free survival over time and account for varying lengths of follow-up. Four a priori categories of 25(OH)D were used: <20 ng/ml, 20–39 ng/ml, 40–59 ng/ml, and ≥60 ng/ml. The 20 ng/ml cut point is from the National Academy of Medicine (NAM, formerly Institute of Medicine) recommendation for bone health [28], the 40 ng/ml cut point is from articles recommending this concentration for the prevention of cancer [29–33], and the 60 ng/ml cut point is from the Lowe et al. study showing reduced breast cancer risk above this concentration [7] and is the top end of the range recommended by a consortium of scientists and physicians to prevent many diseases including breast cancer [29]. Participants were allowed to move between strata of 25(OH)D according to changes in 25(OH)D concentration over the course of the observation periods.

Third, multivariate Cox regression was used to quantify the association between serum 25(OH)D and the risk of breast cancer after adjusting for the following breast cancer risk factors: age, BMI, smoking status, and calcium supplement intake. Indicator variables for study of origin were included to adjust for differences in study methods and demographics. Serum 25(OH)D concentration was assessed as a categorical variable (<20 ng/ml, 20–39 ng/ml, 40–59 ng/ml, and ≥60 ng/ml), as was calcium supplement intake (<1000 mg/day vs ≥1000 mg/day) based on the NAM recommendation for bone health [28]. Serum 25(OH)D concentration and calcium supplement intake changed during the course of the studies for most participants; therefore, these variables were entered as time varying covariates (multiple values were used for each participant to allow for changes in status over time). Age and BMI at baseline were entered as continuous variables and smoking status at baseline was entered as a categorical variable for “current smoker” (yes/no). Since breast cancers diagnosed in the first year were likely present but undiagnosed at study entry, multivariate Cox regression was repeated including only participants free of breast cancer at one year. Additionally, restricted cubic splines with three knots in default locations were used to assess the nature of the association between 25(OH)D (as a continuous variable) and cancer risk, including possible increased risk in the upper serum concentrations. Analyses and graphics were done with the R software (www.r-project.org).

## Results

Baseline demographic characteristics of the pooled and individual cohorts are shown in Table 1. The GrassrootsHealth cohort had a lower median age, BMI, and calcium supplement intake and a lower proportion of participants who were current smokers than either Lappe cohort. The 2007 Lappe cohort had the lowest baseline median serum 25(OH)D concentration (28 ng/ml) and the GrassrootsHealth cohort had the highest (43 ng/ml).

**Table 1:** Characteristics of the pooled, 2007 Lappe, 2017 Lappe, and GrassrootsHealth cohorts.

	Pooled cohort (N = 5038)	2007 Lappe cohort (N = 1129)	2017 Lappe cohort (N = 2196)	GrassrootsHealth cohort (N = 1713)	*P*-value^a^
**Age (years)**: median (IQR^b^)	63 (59–69)	66 (60–71)	63 (59–69)	61 (57–66)	<0.0001
**BMI**: median (IQR^b^)	27 (23–32)	28 (25–32)	29 (25–33)	24 (21–28)	<0.0001
**Smoking status**: N (%)					<0.0001
Current smoker	272 (5%)	104 (9%)	130 (6%)	38 (2%)	
Never or former smoker	4765 (95%)	1025 (91%)	2066 (94%)	1674 (98%)	
**Calcium supplement intake**: median (IQR^b^)	600 (91–1271)	1176 (483–1616)	825 (373–1448)	100 (0–600)	<0.0001
**Serum 25(OH)D (ng/ml)**: median (IQR^b^)					
Baseline	34 (27–43)	28 (23–34)	33 (26–39)	43 (33–58)	<0.0001
Most recent^c^:	38 (29–50)	31 (24–39)	36 (29–46)	49 (37–64)	<0.0001

^a^Statistical comparison of characteristics between the 2007 Lappe, 2017 Lappe, and GrassrootsHealth cohorts. Age, BMI, calcium supplement intake, and serum 25(OH)D concentration were compared using Kruskal-Wallis tests. Smoking status was compared using chi-square test. All risk factors were significantly different (*P*<0.0001) between cohorts and were included in the multivariate Cox regression model to account for these differences.

^b^IQR, interquartile range.

^c^Most recent measurement prior to end of observation (or diagnosis for cases).

During the observation periods, 77 women in the pooled cohort were diagnosed with breast cancer (19 from the 2007 Lappe cohort, 44 from the 2017 Lappe cohort, and 14 from the GrassrootsHealth cohort). The age-adjusted incidence rate of breast cancer was 512 cases per 100,000 person-years in the pooled cohort (458 cases per 100,000 person-years in the 2007 Lappe cohort, 619 cases per 100,000 person-years in the 2017 Lappe cohort, and 337 cases per 100,000 person-years in the GrassrootsHealth cohort).

**Fig 1:**
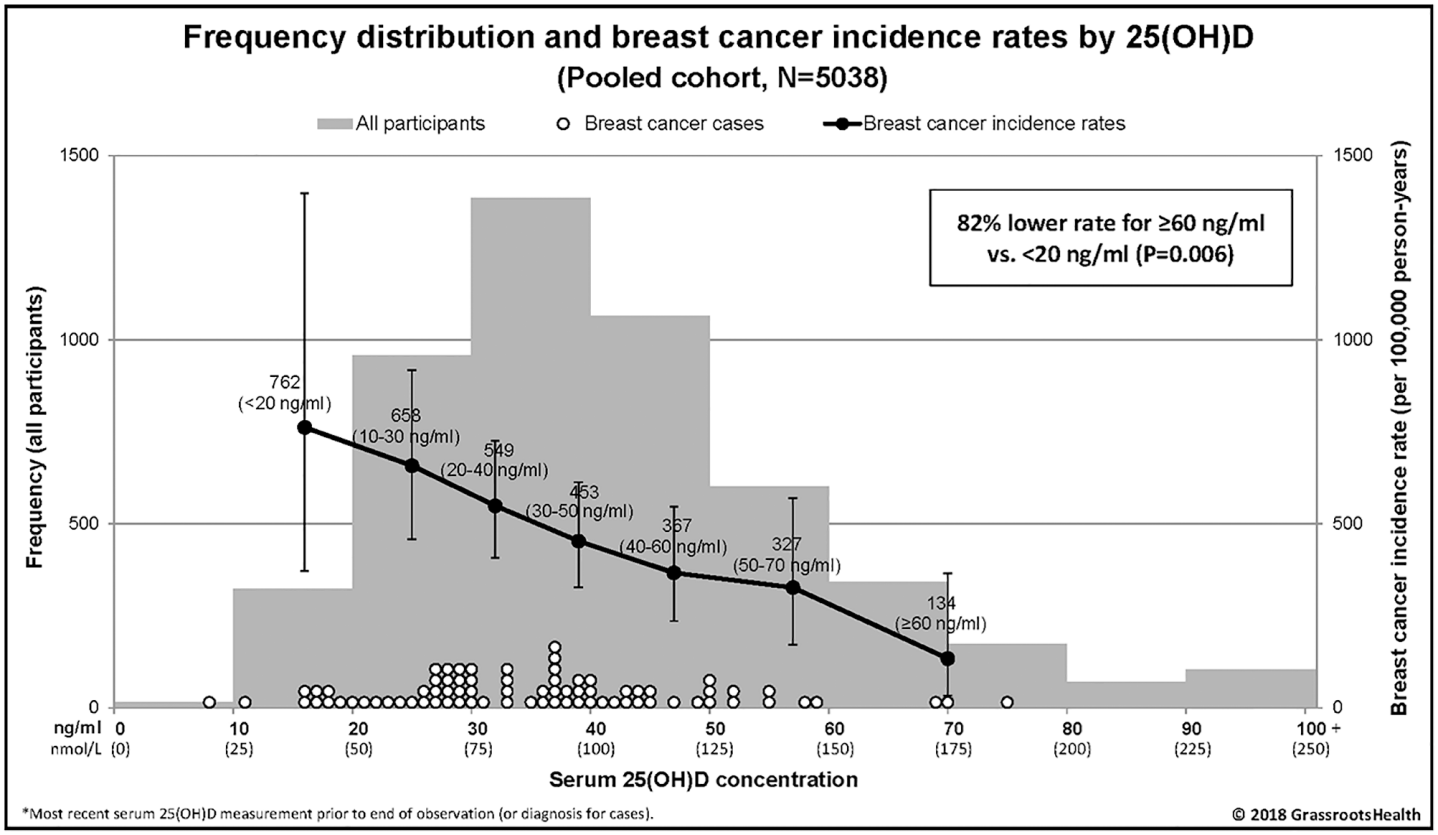
Frequency distribution and breast cancer incidence rates by 25(OH)D concentration, pooled cohort (N = 5038). The bars represent the number of participants by groupings of 10 ng/ml (left y-axis), white dots represent the 25(OH)D concentration for each breast cancer case, black dots represent breast cancer incidence rates per 100,000 person-years for each 25(OH)D group (plotted at the median value for each group: 16, 25, 32, 39, 47, 57, and 70 ng/ml) (right y-axis). Vertical error bars represent the 95% confidence intervals.

Within the pooled cohort, results were similar for the three analyses used to investigate the relationship between 25(OH)D concentration and breast cancer (incidence rate comparison, Kaplan-Meier plot, and multivariate Cox regression). First, breast cancer incidence rates according to 25(OH)D group are shown in Fig 1. Rates were lower with higher serum 25(OH)D categories (Fig 1). Comparing incidence rates, there was an 82% lower incidence rate of breast cancer for ≥60 ng/ml vs <20 ng/ml (Rate Ratio = 0.18, 95% CI: 0.04–0.62, *P* = 0.006).

**Fig 2:**
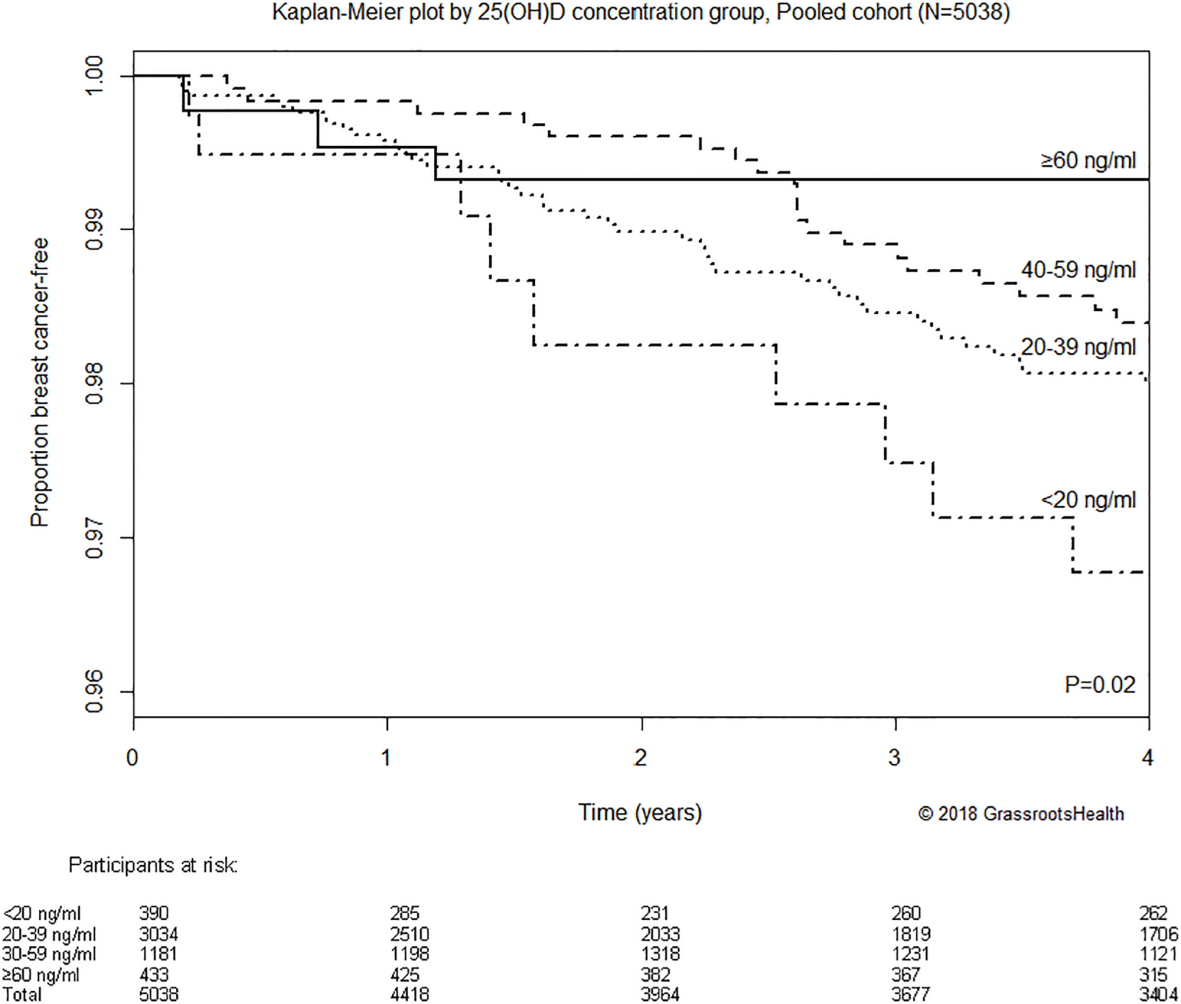
Kaplan-Meier plot comparing the proportion of breast cancer-free participants by 25(OH)D concentration, pooled cohort (N = 5038). Participants were allowed to move between strata of 25(OH)D according to changes in 25(OH)D concentration over the course of the observation periods. Four-year cumulative breast cancer-free proportion was 99.3% among participants with 25(OH)D concentrations ≥60 ng/ml compared to 96.8% for those with 25(OH)D concentrations <20 ng/ml (the proportion with breast cancer was 78% lower for ≥60 ng/ml vs <20 ng/ml, *P* = 0.02).

Second, Kaplan-Meier curves comparing the proportion of breast cancer-free participants by 25(OH)D group are shown in Fig 2. These curves were significantly different (*P* = 0.02), with the highest proportion breast cancer-free at 4 years in the ≥60 ng/ml group (99.3%) and the lowest proportion breast cancer-free in the <20 ng/ml group (96.8%). The proportion with breast cancer was 78% lower for ≥60 ng/ml vs <20 ng/ml (*P* = 0.02) in the Kaplan-Meier analysis.

**Fig 3:**
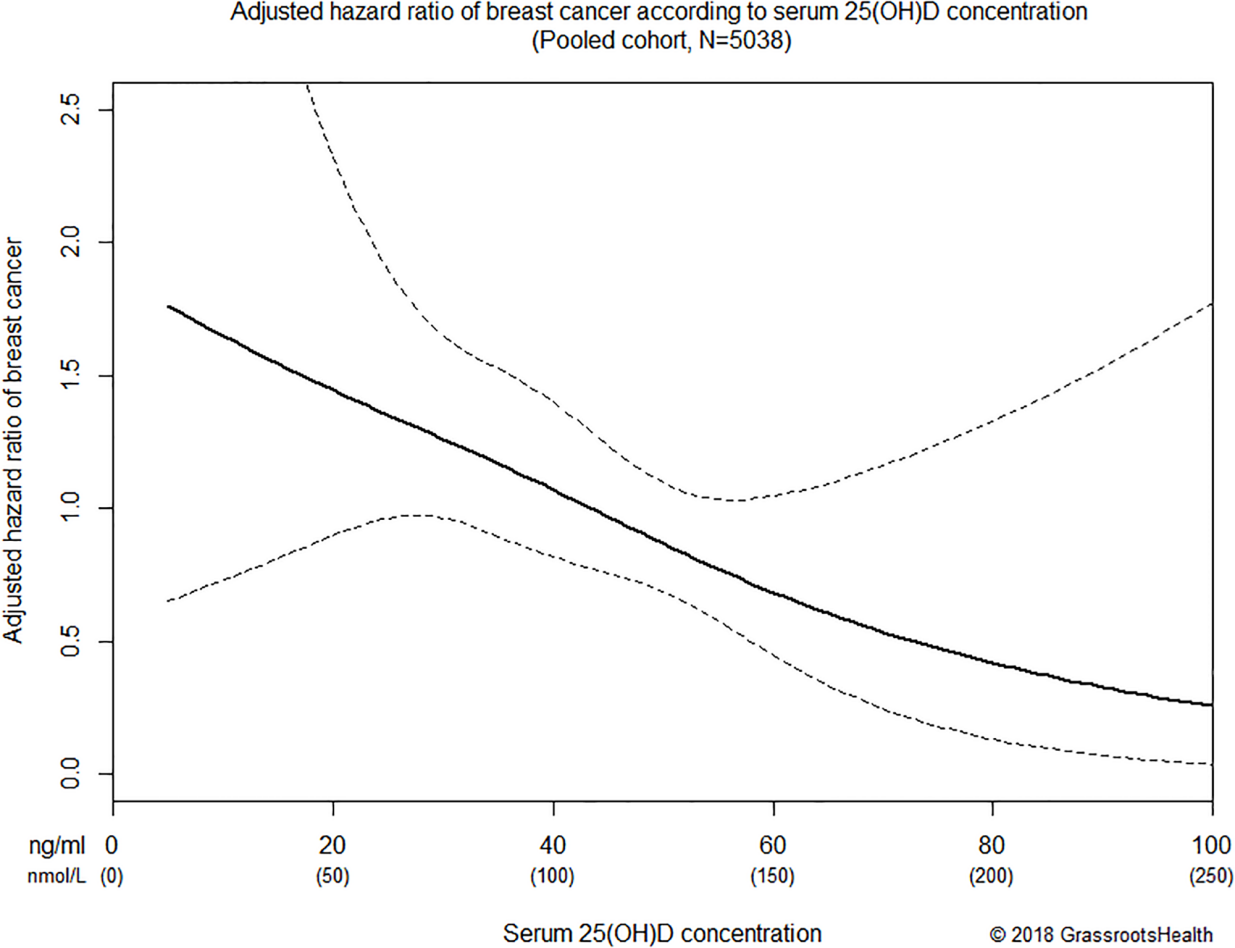
Association between serum 25(OH)D (as a continuous variable) and risk of breast cancer adjusted for age, BMI, smoking status, calcium supplement intake, and study of origin in the range of ≤100 ng/ml, pooled cohort (N = 5308). Solid black line represents the estimated hazard ratio for the Cox regression model with restricted cubic splines with three knots; dashed lines represent the 95% confidence interval of the estimate.

Third, the results of multivariate Cox regression are shown in Table 2 and Fig 3. Women with 25(OH)D concentrations ≥60 ng/ml had an 80% lower risk of breast cancer compared to women with concentrations <20 ng/ml (HR = 0.20, *P* = 0.03), adjusting for age, BMI, smoking status, calcium supplement intake, and study of origin (Table 2). The dose-response decrease in breast cancer risk for women with 25(OH)D concentrations of 20–39 ng/ml and 40–59 ng/ml vs <20 ng/ml are shown in Table 2. Age, BMI, smoking status, calcium supplement intake, and study of origin were not significant predictors of breast cancer risk in this pooled cohort. Among women free of breast cancer at one year (N = 4406), those with 25(OH)D concentrations ≥60 ng/ml had a 93% lower risk of breast cancer compared to women with concentrations <20 ng/ml (HR = 0.07, *P* = 0.02). Spline regression with 25(OH)D as a continuous variable revealed consistently lower risk of breast cancer with higher 25(OH)D concentration, with no evidence of increased risk in the higher 25(OH)D concentrations (Fig 3).

**Table 2:** Association between serum 25(OH)D and risk of breast cancer, pooled cohort (N = 5038).

	Hazard ratio (95% CI), adjusted for study of origin	*P*-value	Hazard ratio (95% CI), adjusted for study of origin and other covariates^a^	*P*-value
Serum 25(OH)D				
<20 ng/ml (<50 nmol/L)	Reference		Reference	
20–39 ng/ml (50–99 nmol/L)	0.61 (0.30,1.26)	0.19	0.55 (0.26,1.16)	0.12
40–59 ng/ml (100–149 nmol/L)	0.52 (0.24,1.16)	0.11	0.48 (0.20,1.14)	0.10
≥60 ng/ml (≥150 nmol/L)	**0.21 (0.05,0.85)**	**0.03**	**0.20 (0.05,0.82)**	**0.03**
*P*-value for trend		**0.03**		**0.04**

Bold values signify significant hazard ratios.

^a^Age, BMI, smoking status, and calcium supplement intake.

Sensitivity analyses were conducted using 25(OH)D concentration quartiles, baseline 25(OH)D concentration only, excluding non-US residents in the GrassrootsHealth cohort, and for each individual cohort. All revealed lower risk of breast cancer with higher 25(OH)D concentration.

## Discussion

In this pooled cohort, 25(OH)D concentration was significantly inversely associated with breast cancer risk. All three analyses showed that women with 25(OH)D concentrations ≥60 ng/ml had significantly lower risk of breast cancer (~80%) compared to women with concentrations <20 ng/ml. There was a consistent decrease in breast cancer risk as 25(OH)D concentrations increased, with no evidence of increased risk in higher concentrations. Using a pooled cohort allowed for analysis across a wider range of serum 25(OH)D concentrations than any of the cohorts alone. While a novel approach, similar inclusion criteria were used for all three cohorts and analyses were adjusted for study of origin and breast cancer risk factors to account for differences in methodology and demographics.

The findings from this analysis support the previously reported inverse association between 25(OH)D and risk of breast cancer [7–18]. Another study assessed breast cancer risk across a broad 25(OH)D concentration range with similar findings [7]. In that hospital-based case control study, Lowe et al. found that women with 25(OH)D concentrations >60 ng/ml had an 83% lower risk of breast cancer than women with concentrations <20 ng/ml (*P*<0.001) [7]. The present study replicated these findings in a much larger, population-based study, thus increasing generalizability, and it’s prospective design enabled use of 25(OH)D values before diagnosis to distinguish between cause and effect.

The Women’s Health Initiative (WHI) trial did not find an association between assigned vitamin D treatment group and breast cancer risk [34]; however, low dosage (400 IU/day) and poor compliance (~50%) likely contributed to the lack of effect. A subsequent re-analysis of the WHI data showed a significant reduction in breast cancer risk among women not taking a vitamin D or calcium supplement before enrollment [35]. A few other nested case-control studies have found no effect [36–38]. Those studies used a single 25(OH)D measurement at enrollment to predict cancer risk over a long follow-up period. That study design does not accommodate changes in vitamin D status over time and diminishes the predictive value of the pre-diagnostic 25(OH)D measurement. Grant has shown that the magnitude and significance level for the relationship between 25(OH)D concentration and breast cancer risk are inversely related to the length of follow-up [39]. In this study, we performed a sensitivity analysis using only baseline 25(OH)D concentration (rather than multiple 25(OH)D values as a time varying covariate) and found a weaker association, also highlighting the diminished predictive value of 25(OH)D concentrations measured long before diagnosis.

Vitamin D may play a number of roles in the prevention of breast cancer development and progression. The biologically active form of vitamin D, 1,25(OH)_2_D3, binds to the vitamin D receptor (VDR) in normal breast epithelium and this complex regulates the cell cycle, promotes differentiation, increases cell-to-cell adhesion, protects cells from DNA damage, regulates cytokines, activates immune cells, and suppresses inflammation, all of which may act to reduce malignant transformations [6]. In breast cancer cells, this complex also activates apoptosis and other mechanisms to suppress tumor growth [6]. Additionally, other vitamin D metabolites from recently discovered alternative pathways, such as 20(OH)D3 from the CYP11A1-mediated metabolism of vitamin D, have been found to have preventive effects similar to 1,25(OH)_2_D3 [40–42]. Studies with respect to cancer treatment have demonstrated vitamin D’s ability to degrade neoplasm [43] and detailed genomics have shown the profound effects vitamin D has on established neoplastic tissue [44]. These mechanisms of vitamin D action provide a possible biological explanation for a causal association between 25(OH)D and breast cancer risk and highlight the importance of assessing this association by the concentration of vitamin D metabolites in the serum and not by indirect measures such as treatment group or supplement intake amount which tend to be inadequate and prone to bias.

Whether our findings reflect prevention of the primary tumor or treatment of early stage, undiagnosed cancer by vitamin D is not clear. Of interest, the results for women who were followed and free of breast cancer at the end of the first year revealed a stronger association between 25(OH)D concentration and breast cancer risk (HR: 0.07, *P* = 0.02 for ≥60 vs <20 ng/ml). There was only one case of breast cancer diagnosed after one year among those with 25(OH)D concentrations ≥60 ng/ml. This woman’s diagnosis occurred 2 months into year two. Since there is a time delay between cancer initiation and diagnosis, many undiagnosed cancers that existed at enrollment would be diagnosed during the first year. Therefore, it is possible that analyses among women free of breast cancer at one year would better assess vitamin D’s specific role in prevention rather than prevention and tumor arrest combined.

While the associations between breast cancer risk and age and calcium supplement intake were in the expected directions (higher risk with increased age and lower risk with higher calcium supplement intake), the effects of these risk factors did not reach statistical significance in this analysis. Since an inclusion criterion for these cohorts was age 55 years and older, the exclusion of younger women may have diminished the effect of age in this analysis. If younger women were included in this study we would expect to see a significant increase in breast cancer risk with age and possibly the effect of age-related changes in vitamin D metabolism. Also, information on dietary calcium intake was not available for the GrassrootsHealth cohort so this analysis only assessed supplemental calcium intake. However, it is possible that dietary calcium intake or total calcium intake would have been a significant predictor of breast cancer risk. Additionally, the small proportion of current smokers may have limited the ability of this study to detect an association between smoking status and breast cancer.

Strengths of this analysis include using a wider range of 25(OH)D concentrations than most other studies and employing multiple analysis techniques with findings of a similar magnitude. Also, using serum 25(OH)D concentration is a better indicator of vitamin D status and statistically more powerful than using treatment group or intake amount because it captures the effect of multiple vitamin D input sources (supplement, sun, and food), overcomes the inherent bias of treatment compliance, and accounts for inter-individual variability in dose response [45]. All three cohorts participated in well-designed population-based studies that included multiple measurements of serum 25(OH)D, allowing for changes in vitamin D status over the course of the observation periods. Using multiple 25(OH)D measurements also overcomes the issue of diminished predictive value of 25(OH)D measurements at enrollment over long follow-up periods. The median amount of time between the 25(OH)D measurement prior to diagnosis and the date of diagnosis was fairly short (~6 months).

Limitations of the analysis include the possible lack of generalizability to younger women and men. However, since other studies have found a significant association between higher 25(OH)D concentrations and lower breast cancer risk in younger women [10–13,18], we would expect that this inverse association is applicable to women of all ages. Also, while there were no ethnic inclusion criteria, the vast majority of participants were non-hispanic white (100% in the 2007 Lappe cohort, 99% in the 2017 Lappe cohort, and 96% in the GrassrootsHealth cohort) so these results may not be generalizable to persons of other ethnicities. While inclusion criteria were matched across cohorts and analyses were adjusted for study of origin and breast cancer risk factors, differences in demographics and methods (e.g. study design, recruitment, and data collection tools) between the cohorts may have affected pooled analyses. Median follow-up time was longer for the Lappe RCTs than the GrassrootsHealth prospective cohort; however, all rate calculations used person-time denominators and analyses accounted for varying lengths of follow-up. Additional limitations include the use of self-reported data and not being able to control for some risk factors (family history of breast cancer, diet, and estrogen use).

The current NAM recommendation of 20 ng/ml (50 nmol/L) is based solely on bone health [28], yet it is widely used as the target level for all health conditions. The findings from this study suggest that breast cancer incidence could be substantially reduced by increasing 25(OH)D concentrations well above 20 ng/ml (50 nmol/L). Fig 3 shows tight confidence bands from about 30 to 55 ng/ml, which represents a decrease in breast cancer risk of ~38%. The high end of that range, 55 ng/ml, falls within the 40 to 60 ng/ml range recommended by a consortium of scientists and physicians to prevent several diseases including breast cancer [29]. Serum 25(OH)D concentrations between 40–60 ng/ml (100–150 nmol/L) are within the physiological range, as evidenced by traditionally living Africans who have a mean 25(OH)D concentration of 46 ng/ml (range: 23–68) with 62% having concentrations between 40–60 ng/ml [46]. In the range of 60 to 100 ng/ml, the downward trend continues. The widened confidence bands stem from the decreasing number of women with 25(OH)D concentrations in this upper range. Clarifying the nature of the association in this upper range should be a high priority for future investigations.

Focusing on primary prevention and implementing evidence-based interventions is needed to substantially decrease breast cancer incidence and associated mortality and economic costs. The national cost of female breast cancer in 2010 was estimated to be $16.5 billion [47]. If women raised their 25(OH)D concentration from their current mean of approximately 30 ng/ml [48] to 55 ng/ml, the analysis from this study suggests that more than $6 billion could be saved every year in the United States. Vitamin D status is a modifiable risk factor for breast cancer, and increasing 25(OH)D concentrations via supplementation at the population level is safe and affordable.
